#  A saúde promovida por redes sociais e comunitárias de mulheres de baixa renda 

**DOI:** 10.1590/0102-311XPT218022

**Published:** 2023-08-11

**Authors:** Renato Luis Barros Lopes, Luis Claudio Ribeiro, Deise Moura de Oliveira

**Affiliations:** 1 Universidade Federal de Juiz de Fora, Juiz de Fora, Brasil.; 2 Universidade Federal de Viçosa, Viçosa, Brasil.

**Keywords:** Rede Social, Comunidades, Determinantes Sociais da Saúde, Mulheres, Social Networking, Communities, Social Determinants of Health, Women, Red Social, Comunidades, Determinantes Sociales de la Salud, Mujeres

## Abstract

As redes sociais e comunitárias constituem um importante determinante social de saúde, especialmente nos segmentos populares da sociedade civil e em suas lutas para garantir o direito à saúde. Esta pesquisa buscou compreender quais são as redes sociais e comunitárias constituídas por mulheres residentes em uma comunidade de baixa renda e sua relação com a produção da saúde nesse grupo social. Foram realizadas entrevistas semiestruturadas com 11 mulheres participantes de uma organização não governamental (ONG) da comunidade, e os dados foram submetidos à análise de conteúdo. A análise destacou quatro categorias: a comunidade como uma grande rede - formada por múltiplas redes dinâmicas interligadas; a rede das “tias” - mulheres com papel importante no cuidado da comunidade, tratadas como parte da família; as rodas de conversa - como o rito de encontro periódico nas calçadas, apontado como importante espaço garantidor de estabilidade emocional e qualidade de vida; e as benzedeiras e o uso de plantas medicinais - referências de cuidado na comunidade, que proporcionam prevenção/tratamento à saúde e encaminhamento para a unidade de saúde quando necessário. Conclui-se que as redes sociais e comunitárias formadas pelas participantes são determinantes sociais importantes. Assim, é necessária a valorização de tais redes pelos equipamentos de saúde inscritos no território.

## Abstract (en)

Social and community networks constitute an important social determinant of health, especially in the poorer segments of civil society and in their struggles to guarantee the right to health care. This study sought to understand the social and community networks created by women living in a low-income community and their relationship with the development of health care in this social group. Semi-structured interviews were conducted with 11 women participating in a nongovernmental organization in the community, and the data were subjected to content analysis. The analysis highlighted four categories: the community as a large network, formed by multiple interconnected dynamic networks; the network of “aunts”, women with an important role in the care of the community, treated as part of the family; the conversation circles, such as the rite of periodic meeting on the sidewalks, indicated as an important space that guarantees emotional stability and quality of life; and the *benzedeiras* with the use of medicinal plants, they are reference care within the community, providing disease prevention, health treatment, and referral to the health care unit when necessary. We concluded that the social and community networks created by the participants are important social determinants, and health facilities registered in the territory should value such networks.

## Resumen

Las redes sociales y comunitarias constituyen un importante determinante social de salud, especialmente en los segmentos populares de la sociedad civil y en sus luchas para garantizar el derecho a la salud. La presente investigación buscó comprender cuáles son las redes sociales y comunitarias tejidas por las mujeres residentes en una comunidad de bajos ingresos y su relación con la producción de la salud en este grupo social. Se realizaron entrevistas semiestructuradas con 11 mujeres participantes de una organización no gubernamental de la comunidad, y los datos se sometieron a análisis de contenido. El análisis destacó cuatro categorías: la comunidad como una gran red - formada por múltiples redes dinámicas interconectadas; la red de las “tías” - mujeres con papel importante en el cuidado de la comunidad, tratadas como parte de la familia; las ruedas de conversación - como el rito de encuentro periódico en las aceras, señalado como un espacio importante que garantiza la estabilidad emocional y la calidad de vida, y las curanderas y el uso de plantas medicinales - referencias de cuidado en la comunidad; que proporcionan prevención/tratamiento a la salud y el encaminamiento para la unidad de salud cuando sea necesario. Se concluye que las redes sociales y comunitarias tejidas por las participantes son determinantes sociales importantes, siendo necesaria la valorización de tales redes por los equipos de salud inscritos en el territorio.

## Introdução

A compreensão contemporânea de saúde, fruto de uma construção histórica, a caracteriza como uma produção social. Nessa perspectiva, depreende-se que a situação de saúde das populações está intimamente relacionada ao contexto em que elas vivem, bem como à posição que ocupam na pirâmide social ^1^. Assim, o contexto de elevada iniquidade em que grande parte da população vive - gerado pelas condições de vida dos distintos grupos, pelas relações de classe e de trabalho, pelo acesso aos serviços de saúde e pelas relações de gênero, étnicas e culturais - repercute no padrão de transmissão de doenças ^2^.

A partir dessa compreensão, emergem os determinantes sociais da saúde (DSS), que são fatores sociais, econômicos, culturais, étnico-raciais, psicológicos e comportamentais que impactam o processo saúde-doença ^3^. O modelo de Dahlgren e Whitehead, um dos mais difundidos e adotados pela comissão nacional dos DSS, concebe as redes sociais e comunitárias como importantes determinantes sociais ^3^, intervindo em fatores coletivos e problemas de saúde, especialmente nos segmentos populares da sociedade civil e em suas lutas para garantir o direito à saúde ^4^.

As redes sociais e comunitárias emergem como um conjunto, ou trama, de relações e intercâmbios entre indivíduos, grupos ou organizações que partilham interesses comuns ^5^, constituindo espaços de trocas coletivas ^6^ que conferem ao sujeito identidade, sentimento e experiências de pertença ^5^. Elas podem ser classificadas como primárias e secundárias. Nas redes primárias, os vínculos estabelecidos são caracterizados pelas relações de parentesco, família, amizade, vizinhança e trabalho e estão fundados sobre a reciprocidade e a confiança. As redes secundárias podem ser formais ou informais e são formadas pelo Estado, mercado e terceiro setor. Os serviços sociais, de saúde ou educacionais são exemplos da rede secundária formal. O terceiro setor, como uma organização não governamental (ONG), constitui exemplo de rede informal ^5^.

Há uma relação estreita entre os DSS e as desigualdades sociais. Nesse sentido, em contextos comunitários de vulnerabilidade, a população feminina merece uma especial atenção. Mulheres negras são marcadas pela violência e pela exclusão. Elas ganham o equivalente a 40% do valor recebido pelos homens brancos ^7^ e são as mais vulneráveis ao desemprego e ao analfabetismo ^8^. São também as que mais sofrem violência - meninas e mulheres negras são 66,7% mais violentadas no Brasil do que mulheres brancas ^8^. Na temática da saúde, a figura da mulher tem forte ligação com o cuidado ^9^. Devido a essa lógica, a mulher, identificada com algumas imagens religiosas de mãe sacrificial, bondosa e altruísta, relaciona-se diretamente com o bem-estar da família, colocando-se em segundo plano em prol da procriação, da família e do casamento ^9^.

Apesar de os DSS serem alvo de estudos nacionais e internacionais ^10^^,^^11^ e o repertório de investigações nesse âmbito ter crescido exponencialmente em todo o mundo ^12^, evidencia-se uma escassez de pesquisas que busquem compreender o determinante social “redes sociais e comunitárias” a partir da realidade vivenciada por mulheres de baixa renda residentes em uma comunidade de alta vulnerabilidade social. Essa lacuna torna-se ainda mais expressiva ao focalizar a compreensão da produção social da saúde a partir de mulheres de baixa renda inseridas em equipamentos sociais pertencentes ao terceiro setor, a exemplo das ONGs. Desse modo, este estudo propõe ampliar o olhar para essa questão ao enfocar mulheres participantes de uma ONG inserida em uma comunidade de alta vulnerabilidade social de um município da Zona da Mata de Minas Gerais.

Essa investigação parte do pressuposto de que a mulher moradora de periferia, a partir de seu contexto, assume um protagonismo no cuidado e na proteção de sua família, além de ter um alto potencial para construir redes, buscando enfrentar os desafios não somente pessoais, mas de toda a comunidade. Diante do exposto, as seguintes questões nortearam a pesquisa: quais redes sociais e comunitárias são tecidas por mulheres residentes em uma comunidade de alta vulnerabilidade social? Como as redes sociais e comunitárias influenciam na produção da saúde de mulheres residentes em uma comunidade de baixa renda? Esta investigação teve como objetivo identificar as redes sociais e comunitárias constituídas por mulheres residentes em uma comunidade de baixa renda e sua relação com a produção da saúde nesse grupo social.

## Método

Este artigo constitui parte dos resultados da dissertação intitulada *Redes Sociais e Comunitárias Tecidas por Mulheres de Baixa Renda Inscritas em uma Organização Não Governamental: A Produção Social da Saúde*^13^, defendida em setembro de 2019 no Programa de Pós-graduação em Saúde Coletiva da Universidade Federal de Juiz de Fora (UFJF).

A pesquisa, de natureza qualitativa, foi realizada em um município localizado na Zona da Mata de Minas Gerais, que tem aproximadamente 570 mil habitantes, com alto Índice de Desenvolvimento Humano Municipal (IDH-M) (0,778). O cenário do estudo foi um bairro de 4.735 habitantes, com claro predomínio da população negra, a qual abrange 69,04% de seus residentes. O bairro teve sua origem na década de 1920, oriundo do processo de decadência do café na região ^14^.

A escolha do cenário da pesquisa deu-se por conveniência, considerando a inserção prévia do pesquisador na comunidade, como diretor de uma ONG que desenvolve projetos de enfrentamento da pobreza, a qual atua em várias frentes, entre elas, a saúde. O estudo teve como participantes mulheres adultas, inscritas em um dos projetos desenvolvidos pela ONG desde 2001, voltado para a saúde da mulher, denominado Vida Plena, o qual conta com a participação de 13 mulheres. Foram excluídas duas participantes que, apesar de pertencerem ao projeto, não moravam mais na comunidade, chegando-se assim a um grupo de 11 mulheres.

A coleta dos dados deu-se em duas etapas. A primeira ocorreu por meio de um questionário estruturado para caracterização do público, aplicado pelo pesquisador em visitas domiciliares, no mês de novembro de 2018. Na segunda, foi feita entrevista semiestruturada, em março de 2019. O pesquisador optou por um ambiente reservado para que as participantes concedessem seus depoimentos. O local foi eleito pelas próprias mulheres, que optaram por realizar as entrevistas em seus próprios domicílios.

O agendamento das entrevistas aconteceu presencialmente, após as reuniões semanais do projeto Vida Plena, do qual todas são participantes. Antes de conceder as entrevistas, as participantes foram informadas quanto aos objetivos do estudo e sobre a importância de sua participação na pesquisa. O Termo de Consentimento Livre e Esclarecido (TCLE) foi lido pelo pesquisador e assinado pelas participantes. Para garantir o anonimato, as participantes foram identificadas com a letra M (de mulher), seguida do algarismo arábico correspondente à ordem de realização da entrevista, a saber: M1, M2, M3… M11. Diante do exposto, as seguintes questões orientaram a entrevista: como é para você ser membro desta comunidade? Como você participa da vida da comunidade? Quais grupos/espaços comunitários você frequenta/faz parte? Como você se sente nesses espaços?

A análise dos dados pautou-se na técnica de análise de conteúdo de Laurence Bardin ^15^. A operacionalização da análise foi realizada conforme as etapas descritas pela autora: pré-análise, exploração do material, tratamento dos resultados obtidos e interpretação. De acordo com os objetivos do estudo, foram definidos os trechos significativos das entrevistas para a posterior elaboração das categorias e subcategorias temáticas, constructo que revela a convergência dos aspectos significativos que emergiram dos depoimentos das participantes. O tratamento dos resultados e sua interpretação dizem respeito ao desvelamento do conteúdo subjacente manifestado na fala das depoentes, somado à interface do mesmo com a literatura pertinente à temática ^15^.

Este estudo obteve parecer favorável do Comitê de Ética e Pesquisa com Seres Humanos da universidade pública do município do estudo (inscrito sob o nº 2.692.001, CAAE 87473018.5.0000.5147).

## Resultado

As participantes tinham média de idade de 47 anos - a mais nova tinha 23 anos e a mais idosa, 74. Do grupo, cinco nunca tiveram a carteira assinada e somente uma tinha essa realidade no momento da entrevista, apesar de três estarem trabalhando. No que tange à escolaridade, duas nunca estudaram, seis tinham Ensino Fundamental incompleto e uma, completo. A renda média das participantes era de R$ 803,54 - a menor renda era de R$ 170,00 e a maior era de R$ 1.600,00. Das 11 mulheres, oito tinham renda *per capita* abaixo da linha da indigência (1/4 do salário mínimo à época = R$ 238,50) e outras duas tinham renda abaixo da linha da pobreza (1/2 salário = R$ 472,50). Somente uma, portanto, estava acima da linha da pobreza.

Sete participantes estavam desempregadas e seis sobreviviam de pensão. Três recebiam Bolsa Família; destas, duas tinham esse benefício como única renda. Dez moravam em casa própria e uma pagava aluguel. Nenhuma das mulheres tinha registro do imóvel em que morava. Somente uma comprou a casa. As demais ganharam o terreno e construíram o imóvel ou já o ganharam construído pela Sociedade de São Vicente de Paulo do bairro - uma organização católica de âmbito internacional, composta por leigos, denominados vicentinos. Eles desenvolvem um trabalho que visa auxiliar o pobre a se emancipar da situação de pobreza em que vive, provendo necessidades básicas como alimentação, moradia e vestuário, com vistas a resgatar a dignidade humana.

### Categoria 1 - a comunidade como uma grande rede

O relato das participantes da pesquisa apresenta o bairro como uma forte teia de relações solidárias, de forma que os moradores estão sempre preocupados com todos: seja em situações de obras, nas dificuldades financeiras, na saúde ou nos conflitos. As mulheres verbalizaram poder contar com essa rede local, que figura como uma rede ativa, sem necessidade de ser acionada pelo demandante. Quem está ao redor é proativo em contribuir, ou seja, não é preciso amizade ou grande afinidade para agir. Ser morador da comunidade e precisar de ajuda são as condições para a rede atuar:

“*No tempo da obra, cada um ficou numa casa. Nessa hora, espalhei todo mundo. Ficaram dois filhos na D.* [amiga] *e dois com a gente lá em cima* [na casa de outra amiga]” (M2).

“...*não tenho que chegar e falar para a pessoa: aqui, se você precisar, você me fala. Não! Se eu estou vendo que você precisa, eu tenho que mostrar meus préstimos*” (M3).

“*Eu usei telefone da D. por 15 anos.* (...) *ligando para São Paulo, Belo Horizonte, Sete Lagoas. Eu pagava, na verdade, mas ela não era obrigada a me emprestar. Eu reconheço isso tudo. Um dia eu cheguei e vi ela com o pé enfaixado. Eu falei: ‘O que que foi, D.?’. Ela falou: ‘Eu torci o pé’* (...). *Ninguém tinha máquina e estava o cesto dela cheio de roupa. Aí falei: ‘Ah, D., eu vou lá em casa e daqui a pouco eu volto aqui para conversar com a senhora’. Vim aqui, troquei de roupa, voltei* (...). *Na mão eu lavei aquela quantidade de roupa. Precisava de banhar o pé dela. O que me custava? Hoje era ela, amanhã podia ser eu. Aí eu fiquei um mês cuidando dela. Ela fala isso até hoje*” (M3).

### Categoria 2 - a rede das tias

Pertencer à comunidade, para as mulheres deste estudo, significa fazer parte de uma grande família. Tal sentimento emerge por causa dos fortes laços de confiança e reciprocidade construídos entre as pessoas da comunidade:

“*Existe o parente de sangue, mas existe o parente madrinha. Ele vira um parente. Tem hora que ele é mais perto que o de sangue. Porque na minha família mesmo de pai e mãe não tinha esse negócio. Se arrumasse filho, se vira. Sai para rua. Não tinha ‘meu pé me dói’, não. Eu vim vivendo e aprendendo isso. Irmão* (...) *era engraçado* (...) *punha pra rua mesmo* (...). *Não tinha ‘meu pé me dói’”* (M2).

“*Qualquer um eu considero parente. Tipo a J. Nós conversamos pra caramba, nós brincamos. A dona L., a N., a F.*” (M4).

“*Eu não tenho parentes aqui, mas eu tenho pessoas que eu posso contar*” (M3).

O sentimento de família, relatado pelas participantes, é expresso pelo afeto e pelos laços construídos longitudinalmente com algumas pessoas da comunidade, que elas tratam e consideram como “tias”, isto é, como parte de suas famílias:

“*Tia L., tia R. Desde pequeninha a gente toma bença à tia R. A gente sempre tomou bença. Tia L., tia R., tia C., irmã da tia L., tia V., irmã da tia L. também. Ali para mim todo mundo é tia. E a gente considera mesmo*” (M7).

“*Gosto muito da tia L.* (...) *eu chamo ela de tia mesmo* (...) *como se fosse. Mas nem é. Eu considero a tia L. como se fosse realmente minha tia*” (M1).

Essas tias desempenham importante papel no apoio à maternidade. Acompanham desde a gravidez, passando pelo parto, até o puerpério. Ensinam, cuidam, receitam chás, ouvem e aconselham quem vive angústias. Devido a isso, têm um importante respeito na comunidade:

“*Dona M. dá banho, cura o umbigo do neném, leva o almoço todo dia e um chá de folha de algodão. Porque o algodão você sabe que é anti-inflamatório. Então, leva durante o resguardo: um mês. Ela ainda ficou com uma menina internada. Eu ia cuidar da outra que ficou aqui. Tinha que dar mamadeira a ela, tinha que dar banho. Tinha que curar o umbigo*” (M3).

“*Nascia uma criança. Às vezes ela mesmo fez o parto. Aí ela tinha que vir* (...) *pegava a banheira, o sabonete, a toalha. Eu falava assim: ‘Mãe, leva a água quente de uma vez. Só tá faltando isso. Ferve a água e leva, porque você tá levando tudo!’*” (M7).

“*Recebi ajuda também na minha última gravidez e na sífilis, quando perdi o neném. A J., A. e a M. Todas me deram um grande apoio*” (M11)

### Categoria 3 - as rodas de conversa

As participantes da pesquisa revelam em suas narrativas a compreensão de que existe uma capilarizada rede dialógica entre elas e a comunidade. Relatam o costume de, ao fim do dia e em especial nos fins de semana, os moradores sentarem em frente às suas casas, na calçada, e passarem horas dialogando. Falam sobre tudo: crises, conflitos e medos, trocando ideias e recebendo conselhos uns dos outros:

“*Isso me faz bem. É muito bom. Uma fala de um problema e outra também. Minha tia, quando fala, ela fica mais leve. O problema some. Quando a gente senta ali é o momento de refletir, desabafar. Como foi a semana inteira. É bom. Você pode perceber que o bairro inteiro é assim. Faz isso*” (M3).

“*Se você olhar pelo emocional, faz muito bem. A gente distrai, esquece os momentos de estresse. Das brigas das crianças* (...). *É o verdadeiro psicólogo de pobre. Porque você relaxa, bate papo, conversa com todo mundo, você fala até o que não quer*” (M1).

“*Aproveito para ficar com a família. Sentamos em frente de casa e ficamos conversando de nossas vidas, nossa luta. São momentos bons. Gosto muito disso. Nem sempre tem bebida, o importante é estar junto*” (M8).

Nesse contexto, projetos como os desenvolvidos pela ONG da qual elas fazem parte servem para fortalecer movimentos já existentes no âmbito comunitário, tornando a troca entre as mulheres uma atividade sistematizada que favorece o intercâmbio de saberes e a criação de vínculos, redes e práticas solidárias mais potentes:

“*Na ONG, as pessoas sabem conversar. Até para falar de fofoca* (...). *Vocês sabem interpretar outras coisas para quem não tem a mente aberta. Ali é um lugar para abrir a mente* (...). *Eu gosto de ir lá*” (M6).

“*Ali, você conversa* (...). *E as meninas dizem: ‘eu fiz isso* (...), *eu fui em tal lugar’* (...) *assim, assim. Foi assim* (...). *Elas te ajudam a resolver coisas que* (...) *você fica ali, igual a uma boba, sem saber o que fazer. Sei lá* (...). *É, é, é bom. É bom. Elas falam muito, mas é bom. Até no seu dia de estresse, você perde o estresse lá. E quando eu entrei para o Vida Plena que eu comecei a ajudar* (...) *e me fez olhar também um pouquinho mais para mim*” (M1).

“*Ele* [o projeto] *é uma reunião que fala sobre qualquer assunto, fala sobre doença, hipertensão, fala sobre qualquer tipo de assunto. Eu gosto. Eu aprendo bastante também* (...). *Adquire mais experiência*” (M4).

“*O Vida Plena é a gente conversar. Juntar as mulheres da comunidade, conversar, ter palestra, ter entendimento, rir um pouquinho. A gente sair um pouco do nosso mundo, sabe?*” (M5).

### Categoria 4 - as benzedeiras e o uso de plantas medicinais

O uso da medicina natural remete a uma questão cultural aprendida pelas mulheres, expressa por elas como uma prática que atravessa gerações e que se materializa em suas experiências cotidianas na/com a comunidade:

“*Porque eu nasci e me criei na roça. Eu saí de lá tinha 21 anos. Foi lá que eu aprend*i” (M3).

“*Faço igual à minha avó. Eu aprendi com ela a usar planta e acho que ela aprendeu com gente do mato*” (M7).

“*Aprendi com meus pais na roça, a gente bebia muito essas coisas. Aí peguei o costume e faço até hoje*” (M10).

O uso da medicina natural está atrelado a alguns nomes de referência na comunidade, como as benzedeiras e estimuladoras da fitoterapia, que são as tias anteriormente descritas:

“*Uso bastante. Chá. Mais do que comprado. Pra gripe, infecção, a gente gosta mais de chá* (...). *Dona M., mãe da N. Porque ela é benzedeira, né? Daqui do bairro. Ela tem muito chá medicinal, muita folha, muita erva. Ela me fala o que que eu tenho que fazer, onde que eu vou pegar. O que ela tem, já junta. Ela é ótima para essas coisas*” (M1).

“*Gosto* (...). *Eu gosto. Não entendo das plantas do mato. A C. sabe. Ela conhece muito mato* (...). *Chá de laranja* (...). *Chá mesmo, assim. Eu tomo porque os outros me dão*” (M2).

“*A dona C., lá em cima, ela está sempre catando chá. Ela receita para os outros. Ela que tem esse negócio do chá. Ela recolhe o chá para as pessoas que pedem pra ela. Ela pega e conhece muito remédio de mato que é bom*” (M10).

Existe uma clara preocupação das benzedeiras com a saúde das pessoas. Apesar de estimularem a prática de plantas medicinais, compreendem que esta não responde a todas as situações de saúde apresentadas pela comunidade:

“*Normalmente, eu uso o chá, se não resolver é que eu levo no médico* (...)” (M1).

“*Tem umas que vem e diz: ‘Ah, eu queria cana de macaco para rins’. Eu falo: ‘Eu, se fosse você, eu ia procurar um médico’. Porque eu sei que o negócio já está feio e chá nenhum vai fazer efeito. ‘Ah, mas eu tomei outro dia que sua mãe me deu* (...) *foi tão bom’. Mas se voltou tão rápido é bom você procurar um médico. Porque às vezes também você vai ficar dando chá, a pessoa vai engambelando com chá e não vai aonde tem que ir*” (M7).

Considerando a relação de grande confiança nessas tias, evidenciou-se que elas são consideradas referências em saúde pelas participantes do estudo, sendo procuradas antes da unidade básica de saúde (UBS) local para a resolução das questões de saúde:

“*Tenho mais confiança nas senhoras daqui do bairro* [do que no médico]. *Ainda mais com o Dr. M*. [médico da UBS]. *Deus que me perdoe. O médico nem olha na nossa cara. Nem olha. Eu, tipo assim, quando estou muito gripada vou na E., que ela tem uma erva, você lava, faz um xarope e bebe. Eu evito ir no médico. Você acorda duas horas da manhã, com esse frio, com chuva e ainda ser maltratado? Eu prefiro ir nessas mulheres e perguntar, que elas falam. É só perguntar a elas que elas sabem de tudo*” (M3).

“*Me sinto mais à vontade com elas* [as benzedeiras]. *Tenho mais intimidade com elas. Porque alguns médicos são educados, mas tem uns que nem na sua cara não olha. Então acaba que a gente não fala*” (M1).

“*Acho que com a D. C. eu falo melhor do que com o médico. Me sinto mais à vontade. Porque o médico enche a gente tanto de pergunta*” (M11).

## Discussão

Uma das marcas do bairro pesquisado é a força das redes sociais locais. O pertencimento e prazer em ser da comunidade são comprovados pela rotina das mulheres, marcada por poucas saídas para outras regiões. Assim, grande parte da rotina, incluindo compras e diversão, ocorre dentro da comunidade. Esse comportamento foi também identificado na pesquisa de Sarti ^16^, que, ao analisar famílias da periferia de São Paulo, identificou uma forte sociabilidade entre os moradores, que minimiza a necessidade e o desejo de sair da comunidade. A tendência a realizar compras na comunidade não ocorre somente pelas facilidades de prazo e deslocamento, mas pelo vínculo criado com os comerciantes locais, o que reforça o sentimento de pertença.

Extrapolando a relação das participantes com o território da comunidade em que vivem, é preciso fazer um contraponto: em que medida essa estreita e intensa relação com o bairro vela a exclusão social que vivem ao morar em uma região periférica de alta vulnerabilidade social? A literatura reporta que, considerando os problemas econômicos, demográficos e sociais, habitar na cidade implica também condições inadequadas de moradia, experiências de exclusão social, reprodução de iniquidades e agravos à população ^17^.

Nesse sentido, pode-se analisar que o uso exponencial do território onde vivem se dá em virtude do não direito à cidade, experimentado por populações que habitam em regiões periféricas/de alta vulnerabilidade social ^17^. Isso acarreta como limitador a dificuldade de acesso aos postos de trabalho e, consequentemente, à renda, em virtude da pouca circulação das pessoas. Esse ciclo retroalimenta a pobreza, configurando um determinante social importante que deve ser considerado ao se investigar populações de baixa renda.

Neste estudo, evidenciou-se também a força da família como agente de proteção e cuidado comunitário, que contrasta com as imagens sociais negativas formuladas sobre famílias pobres. A literatura aponta a tendência de interpretação dessas imagens como desestruturadas e disfuncionais ^17^. Segundo as pesquisadoras, isso ocorre por estereótipos classistas que insistem em atribuir à pobreza o mal social, associando-a à violência, à disfuncionalidade familiar e à degradação moral ^17^. Cabe, portanto, rompermos com esses estereótipos, superando o conceito de funcionalidade atribuído à família, a exemplo das experiências vivenciadas pelas mulheres pesquisadas, que consideram pessoas da comunidade mais família do que as pessoas com as quais têm laços de consanguinidade. Isso demonstra outros sentidos e vínculos fortalecedores dos sentimentos de pertença e de importância do lugar social que ocupam na comunidade.

Uma das bases da qualidade de vida local é a amizade, entendida como conhecer, ser conhecido e respeitado por todos. Amaral ^18^, estudando as relações em periferias, aponta que as amizades têm peso igual ao da família na geração de suporte material e emocional para a sobrevivência. As participantes desta pesquisa têm o costume de nomear pessoas da comunidade como membros da família, como vê-se na figura das “tias” - mulheres de mais idade que exercem forte relação cuidadora e estreito vínculo afetivo com todos da comunidade. Elas atuam em diversos temas, especialmente na saúde: orientam, aconselham, receitam e, em várias situações, cuidam diretamente de moradores da comunidade.

Ao serem analisados conjuntamente o forte vínculo com a comunidade, a relação de amizade entre os moradores, a lógica de uma família extensa ligada por múltiplos laços de parentesco, compadrio e vizinhança, com a marcante vivência da solidariedade na rotina das entrevistadas, nota-se o contexto para a formação de múltiplas redes sociais na comunidade. A literatura coaduna com o que foi evidenciado nesta pesquisa ao afirmar que comunidade é constituída por múltiplas redes, como a dos vizinhos, da família extensa, das madrinhas e tias. Além disso, as condições de inserção não são iguais - nem sempre basta residir na comunidade ^19^. A base dessa rede comunitária é a reciprocidade, ou seja, a possibilidade ou abertura para também agir, ajudar e contribuir quando for possível ^20^.

Segundo Menezes et al. ^21^, a rede social estimula a construção de condutas adaptativas em situações de crise, estresse e doenças. As participantes da pesquisa apontam a rede como produção de alternativas que compensam, ao menos em parte, os efeitos das iniquidades sociais vividas. Dessa maneira, representa caminhos de qualidade de vida e produção de saúde para as mulheres. O impacto das redes sociais e comunitárias é tão grande que seu empobrecimento é um determinante social de saúde tão nocivo como o fumo, a hipertensão, a obesidade e o sedentarismo ^22^.

Ficou evidente na pesquisa que o nível de participação nas múltiplas redes foi singular para cada mulher, a depender de fatores como: tempo de moradia, idade, personalidade, número de familiares residentes no bairro, atitudes de reciprocidade da pessoa, entre outros. Por isso, nem todas as participantes estavam plenamente inseridas nas múltiplas redes e isso influenciou na qualidade de vida relatada por elas ao viver na comunidade.

Um fator importante que a pesquisa evidencia é o uso de plantas medicinais. Esse não é um costume recente, mas derivado da origem rural dos antepassados dessas mulheres. Por isso, não nasce de um simples processo adaptativo local diante dos desafios no acesso a tratamentos e fármacos no Sistema Único de Saúde (SUS). Na comunidade, algumas mulheres são referência no uso de plantas medicinais, devido ao uso próprio de plantas para esse fim e à existência de canteiros em suas casas com algumas variedades. No entanto, o uso da fitoterapia não ocorre somente pela rica diversidade de plantas nas comunidades ^23^, mas, principalmente, por ser o único recurso de que muitas comunidades dispõem e pelo alto custo dos medicamentos ^24^.

É reportado na literatura que em comunidades de baixa renda a coexistência de fatores como a pobreza, a baixa escolaridade, a presença de curandeiros e parteiras, bem como a disponibilidade de plantas medicinais e de derivados vegetais preparados de maneira artesanal - a exemplo das garrafadas -, justifica a prática expressiva da fitoterapia como recurso prioritário de prevenção e tratamento de doenças ^25^.

Há, ainda, as benzedeiras, que adotam uma prática antiga, com somente duas referências na região pesquisada. Elas são grandes promotoras da fitoterapia, pois o ritual de benzeção está sempre interligado ao uso de plantas. Leite & Lima Junior ^26^ apontam o afastamento das benzedeiras dos centros de saber-poder. A marginalização dessas práticas ocorre pela pressão do biopoder em uma lógica de desenvolvimento científico e medicinal. Diante disso, há, muitas vezes, uma inviabilização da atuação das benzedeiras e a construção de um discurso que atribui a elas uma lógica de charlatanismo, demonstrando incompetência cultural e incompreensão e valorização das práticas tradicionais.

Na comunidade, as mulheres que são referência em plantas e as benzedeiras desempenham dois importantes papéis na produção de saúde das participantes da pesquisa. Primeiro, o de prevenção e investimento em saúde, pois elas multiplicam orientações e práticas de prevenção a diversas enfermidades e agravos de saúde; e segundo, o de atuarem no primeiro passo de cuidado das enfermidades básicas da população. A postura de uma benzedeira demonstrou não haver separação entre o uso dos fitoterápicos e o acesso à UBS. Ela apontou que quando identifica que o uso das plantas não atendeu à queixa da pessoa, deixa de fornecer a matéria-prima e a orienta a buscar atendimento na UBS.

As entrevistadas, por sua vez, apontaram buscar primeiro as benzedeiras e utilizar prioritariamente as plantas medicinais para o tratamento de diversas enfermidades, devido ao vínculo e à confiança que têm nas mulheres de referência. Apenas quando as práticas tradicionais não atendem à demanda é que as participantes buscam o sistema formal de saúde. As mulheres da pesquisa foram categóricas em afirmar que se sentem mais acolhidas ao relatar os sinais e sintomas às benzedeiras, sentindo-se melhor compreendidas e orientadas por essas referências de cuidado na comunidade.

As entrevistas apontam que um espaço significativo na rotina das mulheres foi constituído pelo bate-papo e pela convivência na calçada, que deixou de ser um simples local público de trânsito na comunidade. Sentadas ao chão, elas se abrem, ouvem conselhos, refletem sobre seus desafios, atualizam as informações da comunidade e confraternizam com comida e bebidas - muitas relatam alívio de estresse após esses momentos. Há, inclusive, datas semanais fixas para ocorrer esses eventos, referidos pelas entrevistadas como “psicólogo de pobre” ou “postinho”, em alusão à UBS. Segundo Mello ^27^, as rodas de conversa geram espaços de diálogo e escuta, favorecendo a compreensão crítica sobre a realidade da qual o ser humano é agente. São espaços de cuidado próprio, a partir do movimento de voltar seu olhar para si, examinando as próprias histórias de sua existência. A alusão a um profissional e a um equipamento de saúde aponta como esse ambiente contribui para a produção social da saúde dessas mulheres e, por isso, tem um significado tão importante em sua rotina.

Ao estudarem o espaço público e privado nas favelas, Peregrino et al. ^28^ indicam que o tecido urbano complexo de regiões com moradias muito pequenas e próximas e vias subdimensionadas cria espaços de sociabilidade em que público e privado se confundem. Há, então, o cenário em que as ruas são vistas como continuação das casas e tornam-se espaços semiprivados, ao passo que a maioria dos terraços das casas pode constituir espaços semipúblicos.

Esta pesquisa tem como limitação o critério de escolha das participantes, pois as mulheres integrantes do projeto social de uma ONG foram selecionadas por conveniência do entrevistador, o que caracteriza uma rede social informal da comunidade. Assim, não houve participação de nenhuma mulher fora da rede, o que permitiria uma melhor análise dos resultados. Além disso, o pesquisador tem laços antigos com as participantes, com algumas há mais de dez anos. Adicionalmente, é homem, branco e diretor da instituição de que as mulheres participam. Isso, sem dúvidas, interferiu nas respostas das participantes.

## Conclusão

Esta pesquisa buscou compreender como as redes sociais e comunitárias influenciam na produção da saúde de mulheres de baixa renda atendidas por uma ONG. Foram identificadas múltiplas redes sociais, como a de vizinhos, amigos, madrinhas/tias e benzedeiras, que constituem redes dinâmicas, as quais se interligam e formam complexos mosaicos.

Evidenciou-se que essas redes estão em constante movimento de construção e reconstrução. Não basta morar no bairro para estar inserido nelas, pois existem níveis de pertença diferenciados. É preciso desejo e investimento da pessoa. As múltiplas redes solidárias num contexto de fortes laços acabam gerando uma grande e extensa família, sendo sua marca mais simbólica as “tias”, interpretadas neste estudo como “mães coletivas” que ajudam, protegem e cuidam de todos.

As redes sociais e comunitárias locais são potencializadas pelas instituições presentes no bairro, incluindo a ONG da qual as mulheres participam. Os projetos da ONG estimulam o fortalecimento das redes já presentes na comunidade, por meio de uma convivência qualificada e da minimização de seus fatores de risco. Ademais, propiciam melhores condições de habitação e empregabilidade para as mulheres.

Destacam-se, ainda, as redes tecidas no cotidiano, como no rito de encontro periódico nas calçadas, que foi apontado como espaço significativo para estabilidade emocional e qualidade de vida das mulheres e comparado, pelas participantes, à ação de um profissional de saúde e de equipamentos como a UBS. Por fim, existe o costume do uso de fitoterápicos estimulado pelas benzedeiras e mulheres de referência em plantas. Essas líderes são referência na produção social de saúde para as mulheres, especialmente diante dos desafios de acolhimento e ambiência da UBS.

Os achados desta investigação identificaram que as redes sociais e comunitárias tecidas pelas mulheres participantes da pesquisa são importantes DSS que atuam de modo a promover saúde no grupo pesquisado. Elas conferem às mulheres suporte material e emocional para a qualidade de vida, atuando direta e/ou indiretamente na prevenção de morbidades, promoção de cuidados e saúde emocional.

Esta pesquisa aponta para a importância de os equipamentos de saúde visibilizarem, valorizarem e potencializarem as redes sociais e comunitárias em regiões de especial interesse social, pois elas podem ser importantes aliadas na produção social de saúde de uma comunidade. Além disso, são necessários estudos que busquem compreender como essas redes são constituídas em diferentes contextos da sociedade - considerando o não direito à cidade vivenciado por populações de baixa renda -, o que pode revelar resultados distintos dos encontrados nesta investigação.
